# Antimicrobial Activity of Six Pomegranate (*Punica granatum* L.) Varieties and Their Relation to Some of Their Pomological and Phytonutrient Characteristics

**DOI:** 10.3390/molecules14051808

**Published:** 2009-05-13

**Authors:** Ahmet D. Duman, Mustafa Ozgen, Kenan S. Dayisoylu, Nurcan Erbil, Coskun Durgac

**Affiliations:** 1 Department of Food Engineering, University of Kahramanmaras Sutcu Imam, 46060, Kahramanmaras, Turkey; E-mail: kesiday@ksu.edu.tr (K.S.D.); 2 Department of Horticulture, University of Gaziosmanpasa, Tasliciftlik, 60240, Tokat, Turkey; E-mail: mozgen@gop.edu.tr (M.O.); 3 Department of Biology, University of Kahramanmaras Sutcu Imam, 46060, Kahramanmaras, Turkey; E-mail: nurcanerbil@hotmail.com (N.E.); 4 Department of Horticulture, University of Mustafa Kemal, 31040 Antakya, Turkey; E-mail: cdurgac@mku.edu.tr (C.D.)

**Keywords:** antimicrobial bioactivity, anthocyanin, phenolics, pomegranate, *Punica granatum* L.

## Abstract

Arils from six pomegranate (*Punica granatum* L.) varieties grown in the Mediterranean region of Turkey were tested for their antimicrobial properties by the agar diffusion and minimum inhibitory concentration (MIC) methods against seven bacteria: (*Bacillus megaterium* DSM 32, *Pseudomonas aeruginosa* DSM 9027, *Staphylococcus aureus* Cowan 1, *Corynebacterium xerosis* UC 9165, *Escherichia coli* DM, *Enterococcus faecalis* A10, *Micrococcus luteus* LA 2971)*,* and three fungi (*Kluvyeromyces marxianus* A230, *Rhodotorula rubra* MC12, *Candida albicans* ATCC 1023). It has been observed that the pomegranate aril extracts had antimicrobial effect on all microorganisms, giving inhibition zones ranging in size from 13 to 26 mm. The MIC values for active pomegranate extracts ranged between 30 and >90 µg/mL. The results obtained appeared to confirm the antimicrobial potential of the *Punica granatum* varieties.

## Introduction

The pomegranate, *Punica granatum* L., is one of the oldest known edible fruits. This fruit is mentioned in the Bible and Koran and is often associated with fertility. It is native to Persia and from there it spread into Asia, North Africa and Mediterranean Europe, including Turkey. The domestication process took place independently in various regions. In Turkey planting of pomegranates has increase rapidly in recent years and the total production in 2007 exceeded 100,000 tons [1].

*Punica granatum* L. has been widely used by traditional medicine in America, Asia, Africa and Europe for the treatment of different types of diseases [2,3,4,5]. In the ancient Egyptian culture the pomegranate fruit was regarded as a symbol of prosperity and ambition, making it common practice to decorate sarcophagi with depictions of the plant. According to Eber’s Paprus (one of the oldest medical writings, dated *circa* 1,500 BC), the plant was used by the Egyptians as a treatment for tapeworm and other parasitic infestations [5]. The fruits of *Punica granatum* (pomegranate) have been used to treat acidosis, dysentery, microbial infections, diarrhoea, helminthiasis, haemorrhage, and respiratory pathologies [6]. Furthermore, this species appear to have interesting antiviral activity. Pomegranate extracts have been shown to be effective against the herpes virus [7] and hydroalcoholic extracts of whole fruits have exhibited high activity against the influenza virus [8,9]. 

The antimicrobial activity of some of the common pomegranate cultivars has also been studied [10,11]. Braga *et al*. [12] showed that pomegranate extracts inhibit and delay *Staphylococcus aureus* growth and subsequent enterotoxin production at 0.01, 0.05 and 1% v/v concentrations. Melendez and Capriles [13] have also reported that extracts from *Punica granatum* fruits possess strong *in vitro* antibacterial activity against many bacterial strains tested. 

In recent years, there has been an increasing interest in determining antioxidant properties of red fruits, due to their rich dietary sources of antioxidant phenolics and anthocyanins [1,14,15]. The goal of this study was to investigate the antimicrobial activity of some of the new popular pomegranate cultivars grown in Turkey. We also aimed to evaluate the correlations between responses by microorganisms and pomological and phytonutrient properties such as total phenolic content (TP), total antioxidant capacity determined by TEAC (Trolox Equivalent Antioxidant Capacity) and FRAP (Ferric Reducing Antioxidant Power).

## Results and Discussion

The pomological and antioxidant properties of the same pomegranate fruit samples used in this study were previously determined and published in Ozgen *et al*. [1]. A summary of these characteristics are presented in Table 1 to allow evaluation of possible correlations with the antimicrobial activities seen in this work.

The extracts obtained from fruits of six popular pomegranate cultivars were found to be effective against the bacteria *B. megaterium*, *P. aeruginosa*, *S. aureus*, *C. xerosis*, *E. coli*, *E. faecalis* and *M. luteus*, showing inhibition zones ranging from 13-26 mm (Table 2). 

There were significant differences between the inhibition effects on different microorganisms. For example, the highest inhibition was obtained for *E. coli*, while the lowest was observed for *E. faecalis*. The inhibition responses also varied among pomegranate cultivars. Katirbasi and Tatli, the least acidic and displaying the lowest amount of total monomeric anthocyanin (TMA), TP, TEAC and FRAP, were less effective in inhibiting microorganisms, compared to the other tested cultivars. Indeed, Katirbasi was only effective against *E. coli*, while Tatli has an effect on *R. rubra*. Serife, which is the most acidic cultivar and has the second highest phenolic content also had the greatest inhibitory effect and its inhibition zones averaged from 25 mm (on *E. coli*) to 17 mm (on *R. rubra*). Eksi was the second most effective inhibitory cultivar with an average of 19.4 mm. Its effect against *E. coli* was the highest among all cultivars tested. Kan had an average of 17.8 mm inhibition and it was more effective against *P. aeruginosa*, *C. xerosis* and *E. coli*, compared to other microorganisms. Although Dikenli Incekabuk had no effect on *C. albicans*, the bioactivity for other microorganisms ranged between 15.3 to 23.3 mm, with an average of 17.2 mm.

The antimicrobial effects of pomegranate were previously studied. Indeed, it is reported that the bark, leaves, flowers, and fruits of pomegranate are widely used as phytotherapeutic agents in Brazil [25]. Ahmad and Beg [26] reported that alcohol extracts of pomegranate fruits showed antibacterial activity when tested against *S. aureus*, *E. coli* and *Shigella dysenteriae*. Prashanth *et al.* [27] also reported methanolic extracts of *Punica granatum* fruit rind to be active against all microorganisms tested in their study. Mathabe *et al*. [25] showed that methanol, ethanol, acetone, and water extracts obtained from pomegranate were active and effective against the tested microorganisms (*S. aureus, E. coli, Salmonella typhi, Vibrio cholera, S. dysenteriae, S. sonnei, S. flexneri, S. boydii*), showing an inhibition zones of 12-31 mm. Melendez and Capriles [15] have also reported that extracts from pomegranate fruits possess strong *in vitro* antibacterial activity against many bacteria tested (*E. coli*, *Enterobacter cloacae*, *P. fluorescens*, *Proteus vulgaris*, *Alcaligenes faecalis*, *Serratia marcescens*, *E. aerogenes*, *S. aureus*, *Arthrobacter globiformis*, *M. luteus*, *B. cereus*, *B. subtilis*, *B. coagulans*, *Micrococcus roseus*, *M. phlei*, *M. rodochrus*, *M. smegmatis*; showing an inhibition zones of 11-31 mm). Interestingly, they stated that in Puerto Rico, it is a very common practice to use these plant extracts as remedies for colds and bacterial infections. Their results provide evidence for the presence of antimicrobial compounds in the crude methanolic extracts of these plants. These findings and our result clearly demonstrated and confirmed the effectiveness of pomegranate fruit on inhibition of microbial activity. 

The minimal inhibitory concentration (MIC) values for bacteria (*B. megaterium*, *P. aeruginosa*, *S. aureus*, *C. xerosis*, *E. coli*, *E. faecalis* and *M. luteus)* and fungi (*K. marxianus*, *R. rubra* and *C. albicans)* were determined as evaluation of the antimicrobial activity of the pomegranate cultivars and presented in Table 3. Kan, Serife, Eksi and Dikenli Incekabuk extracts revealed higher antimicrobial activity at low concentrations against bacteria and fungi, compared to the Katirbasi and Tatli cultivars. The MIC values for active pomegranate extracts ranged between 30 and >90 µg/mL. The antimicrobial activity of phenolic compounds is well documented [15,28,29,30,31]. Food extracts may be more beneficial than isolated constituents, due to the other compounds present in the extracts can change the properties of bioactive individual component [32]. 

In general, the extent of the inhibitory effects of the pomegranate extracts could be attributed to their phenolic and anthocyanin content of fruits. The bioactivity of aril extracts on the microorganisms tested has high total flavonols, phenolics, anthocyanins and organic acids. Similarly, Shoko *et al*. [33] confirmed that phenolics were the most important compounds against bacteria, among those gallic acid was identified as the most active compound for inhibition of bacteria tested. Our findings are also support these results. The inhibitory effect of phenolic compounds could be explained by adsorption to cell membranes, interaction with enzymes, substrate and metal ion deprivation [34]. These results confirmed the antibacterial potential of pomegranate and its use in traditional medicine [25].

Different from most of the previous studies, we investigated the correlation between several pomological and antioxidant characteristics and antimicrobial activity of pomegranate fruit extracts (Table 4). Among the peel color measurements, “L” and “b” values and acidity were found to be not correlated with inhibition zones for any of the microorganisms tested. “Chroma” and “a” values had the same patterns for correlations and they were significantly correlated with inhibition effects against most microorganisms other than *E. coli*, *R. rubra* and *C. albicans*. High “Chroma” and “a” values indicated high red color, and in consequence high anthocyanin content. Brix and pH was found to be correlated with the inhibition effect against *E. faecalis*. TP, TMA, TEAC, and FRAP were exhibited significant correlation with the inhibitory effects against all the microorganisms except *R. rubra* and *C. albicans*. It is known that TP, TMA, TEAC, and FRAP are usually well correlated in red color-anthocyanin rich fruits [23,24]. In our study, especially highly acidic and anthocyanin rich, dark red color cultivars displayed high inhibitory effects. 

## Conclusions

The results obtained from this study showed that new popular pomegranate cultivars grown in the Mediterranean region of Turkey have antibacterial and antifungal potential. More importantly, the results indicated that pomegranate cultivars high in acid and rich in phenolics and anthocyanins have higher antibacterial and antifungal activity and this is closely correlated with pomegranate antioxidant capacity. 

## Experimental

### Plant material and preparation of fruit extract

Commercially ripe fruits from six pomegranate cultivars (Dikenli Incekabuk, Eksi, Kan, Katirbasi, Serife and Tatli) were harvested from various places of Mediterranean region of Turkey. Arils of fruits (about 100 g lots) were hand-separated and frozen at -20 °C. Three replicates were maintained for each analysis, each replicate composed of six pomegranate fruits. Arils from each cultivar were thawed at room temperature and then homogenized in a food processor. Slurries were used to prepare fruit extracts for antimicrobial activity tests.

### Antimicrobial activity

*Microbial cultures*: Cultures of *B. megaterium* DSM 32, *P. aeruginosa* DSM 9027, *S. aureus* 6538, *C. xerosis* UC 9165, *E. coli* DM, *E. faecalis* A10, *M. luteus* LA 2971, *K. marxianus* A230, *R. rubra* MC12, *C. albicans* ATCC 1023 were used in our study. These microorganisms were provided from culture collections of the Microbiology Laboratory of Science and Art Faculty of the University of Kahramanmaras Sutcu Imam, in Turkey. 

*Preparation of microorganism cultures*: The above-mentioned bacteria were incubated at 37±0.1 ^o^C for 24 h by injection into Nutrient Broth (Difco), and the studied mould and yeasts were incubated in Sabourand Dextrose Broth (Difco) for 24 h. Mueller Hinton Agar (MHA-Oxoid) and Sabourand Dextrose Agar (SDA) sterilized in a flask and cooled to 45-50 ^o^C were distributed to sterilized petri dishes having a diameter of 9 cm (15 mL) after injecting cultures (0.1 mL) of bacteria and yeasts (10^6^-10^7^ bacteria per mL and 10^6^ yeasts per mL), NCCLS [16], and distributing medium in Petri dishes homogenously [17,18]. 

*Determination of antimicrobial activity:* The agar diffusion method was used to detect the antimicrobial activities of the extracts. Four equidistant holes were made in the agar using sterile cork borers (No.9, Ø 11 mm). A volume of pomegranate extract (100 µL) was added to the holes using a pipettor [21]. Petri dishes were placed at 4 ^o^C for 2 h, the dishes injected with yeasts were incubated at 25±0.1 ^o^C and bacteria were incubated at 37 ^o^C for 24 h [19,20]. At the end of the period, inhibition zones formed on the MHA and SDA (measured in mm) were evaluated. Studies were performed in triplicate, and the developing inhibition zones were compared with those of reference antibiotics (streptomycin, tobramycine, and nystatin).

*Microbiological numbers:* Microbial numbers were determined by standart plate counts, using plant count agar and potato dextrose agar (Difco Laboratories). Plates were incubated at 25, 37 ^o^C and colonies arising after 24-72 h were counted [19].

*Determination of minimal inhibitory concentration (MIC):* A broth micro-dilution susceptibility assay was used, as recommended by NCCLS, for the determination of the MIC of pomegranate extracts and some reference components [16]. All tests were performed in MHB supplemented with Tween 80 detergent [final concentration of 0.5% (v/v)], with the exception of the yeasts (SDB+Tween 80). Bacterial strains were cultured overnight at 37 ^o^C in MHB, and the yeasts were cultured overnight at 25 ^o^C in SDB. Geometric dilutions ranging from 20, 30, 40, 50, 60, 70, 80, 90 and 100 µg/mL of the pomegranate extracts were prepared including one growth control (MHB+Tween 80) and sterility control (MHB+Tween 80+test extract). Test tubes were incubated under normal atmospheric conditions at 37 ^o^C for 24 h for bacteria and at 25 ^o^C for 48 h for the yeasts. The bacterial growth was indicated by the presence of a white “pellet” on the well bottom.

### Statistical analysis

All experiments were carried out at least in triplicate and statistical analysis was performed using SAS procedures [22]. Pomological, antioxidant and antimicrobial activity analysis were performed with using the same fruit or fruit extracts. DIZ (Diameter Inhibition Zones) and MIC data were first analyzed by Analysis of Variance (ANOVA) using GLM procedure. CORR was used to determine correlation coefficients between DIZ/MIC data and several fruit characteristics which were previously published [1]. 

## Figures and Tables

**Table 1:** Pomological and phytonutrient characteristics of the pomegranate cultivars studied. Data adapted from a previous study by Ozgen *et al*. [1].

Genotype	^a^L	^a^a	^a^b	^a^Chroma	*^a^hue*	^b^TSS	^c^Acidity	pH	^d^TP	^e^TMA	^f^TEAC	^g^FRAP
Dikenli Incekabuk	70.5	18.4	34.1	41.0	63.1	16.9	1.88	2.71	1395	38.24	5.84	7.84
Eksi	73.6	3.9	39.2	40.6	83.8	16.7	1.70	2.88	1465	37.53	5.33	7.52
Kan	57.3	35.0	30.4	46.6	41.7	17.3	1.92	2.94	2076	218.93	7.70	10.92
Katirbasi	74.3	1.6	40.8	41.6	87.4	15.9	1.18	3.14	1326	41.23	4.38	5.37
Serife	73.7	17.5	29.8	37.4	62.6	15.8	2.98	2.75	1532	18.01	5.64	7.80
Tatli	67.0	15.3	34.6	38.9	66.4	15.4	0.42	3.15	1245	6.12	4.73	4.63

^a^Aril color was measured a Minolta chromameter (model CR-400; Minolta, Ramsey, NJ, USA) which provided CIE L*, a* and b* values. These values were used to calculate chroma; which indicates the intensity or color saturation, and hue angle; ^b^TSS (Total soluble solids) values are expressed as %Brix; ^c^Acidity, values are expressed as %; ^d^TP (Total phenolic) values are expressed as mg gallic acid equivalents (GAE)/L juice; ^e^TMA (Total monomeric anthocyanin) values are expressed as mg cyanidin 3-glucoside equivalents/L juice; ^f^TEAC (Trolox equivalent antioxidant capacity) values are expressed as mmol of Trolox equivalents/L juice and ^g^FRAP (Ferric Reducing Ability of Plasma) values are expressed as mmol of Trolox equivalents (TE)/L juice.

**Table 2:** Inhibition zones (mm) on several microorganisms caused by six pomegranate cultivars and some positive antibiotics exhibiting their antimicrobial effects.

Microorganisms	Cultivars							Reference antibiotics/DIZ^a^		
	Kan	Katirbasi	Tatli	Serife	Eksi	Dikenli Incekabuk	Mean	S10^b^	T10^c^	N100^d^
*Bacillus megaterium*	18.0	0.0	0.0	20.0	17.7	18.7	12.4 E	17	16	nt^†^
*Pseudomonas aeruginosa*	20.3	0.0	0.0	19.3	20.7	21.0	13.6 C	14	12	nt
*Staphylococcus aureus*	12.7	0.0	0.0	20.3	17.7	15.7	11.1 G	17	13	nt
*Corynebacterium xerosis*	21.7	0.0	0.0	20.3	14.0	19.3	12.6 E	15	13	nt
*Escherichia coli*	23.0	21.3	0.0	25.0	26.0	22.7	19.7 A	-	10	nt
*Enterococcus faecalis*	12.7	0.0	0.0	24.0	15.7	23.3	12.6 E	17	12	nt
*Micrococcus luteus*	15.0	0.0	0.0	17.7	20.7	16.3	11.6 F	-	-	nt
*Kluvyeromyces marxianus*	18.3	0.0	0.0	24.3	16.3	19.7	13.1 D	nt	nt	13
*Rhodotorula rubra*	15.3	0.0	16.7	17.0	22.0	15.3	14.4 B	nt	nt	14
*Candida albicans*	20.7	0.0	0.0	23.3	23.7	0.0	11.3 H	nt	nt	18
Mean	17.8 c*	2.1 e	1.7 f	21.1 a	19.4 b	17.2 d	13.2			

*Small letters compare the pomegranate cultivars while capital letters compare the microorganisms, Means not followed by the same letter are significantly different at *P* < 0.05 according to the Least Significance Difference (LSD) mean separation method, ^a^Antibiotic discs/Diameter of inhibition zone (mm); ^b^Streptomycin: 10 µg/disc; ^c^Tobramycine: 10 µg/disc; ^d^ Nystatin: 100 U/disc; ^†^Not tested.

**Table 3:** Minimum inhibitory concentration of pomegranate cultivars on some microorganisms.

Microorganisms	cfu^a^/mL inoculum	Minimal Inhibitory Concentrations (MIC- µg/mL)					
		Cultivars					
		Kan	Katirbasi	Tatli	Serife	Eksi	Dikenli Incekabuk
*Bacillus megaterium*	10^7^	40	>90	>90	40	40	40
*Pseudomonas aeruginosa*	10^7^	40	>90	>90	40	40	40
*Staphylococcus aureus*	10^7^	50	>90	>90	40	40	40
*Corynebacterium xerosis*	10^7^	40	>90	>90	40	50	40
*Escherichia coli*	10^7^	40	30	>90	30	30	30
*Enterococcus faecalis*	10^7^	40	>90	>90	30	40	30
*Micrococcus luteus*	10^7^	50	>90	>90	40	40	40
*Kluvyeromyces marxianus*	10^6^	40	>90	>90	40	40	40
*Rhodotorula rubra*	10^6^	50	>90	40	40	40	40
*Candida albicans*	10^6^	30	>90	>90	40	40	>90

^a^Number of Colony Forming Units.

**Table 4:** Correlation coefficients of inhibition zones on several microorganism caused by six pomegranate cultivars and their fruit characteristics. Significant correlations are bolded at p≤0.05.

Microorganisms	L	a	b	Chroma	*hue*	TSS	Acidity	pH	TP	TMA	TEAC	FRAP
*Bacillus megaterium*	0.35	**0.56**	0.11	**0.54**	**0.49**	-0.38	-0.20	0.22	**0.69**	**0.66**	**0.64**	**0.68**
*Pseudomonas aeruginosa*	0.41	**0.58**	0.15	**0.55**	**0.52**	-0.44	-0.15	0.14	**0.68**	**0.67**	**0.59**	**0.64**
*Staphylococcus aureus*	0.29	**0.57**	0.05	**0.56**	0.45	-0.37	-0.15	0.34	**0.65**	**0.63**	**0.67**	**0.67**
*Corynebacterium xerosis*	0.28	**0.52**	0.05	**0.50**	0.44	-0.32	-0.31	0.18	**0.71**	**0.62**	**0.61**	**0.67**
*Escherichia coli*	0.46	0.37	0.32	0.33	0.36	-0.46	-0.06	0.05	**0.90**	**0.97**	**0.87**	**0.76**
*Enterococcus faecalis*	0.32	**0.69**	0.05	**0.66**	**0.63**	-0.51	-0.44	**0.54**	**0.71**	**0.62**	**0.74**	**0.81**
*Micrococcus luteus*	0.39	**0.56**	0.15	**0.54**	**0.47**	-0.40	-0.02	0.16	**0.62**	**0.66**	**0.59**	**0.60**
*Kluvyeromyces marxianus*	0.28	**0.57**	0.01	**0.57**	0.46	-0.34	-0.31	0.34	**0.71**	**0.63**	**0.69**	**0.72**
*Rhodotorula rubra*	0.07	-0.33	-0.09	-0.34	0.19	-0.01	0.21	0.09	0.11	0.08	0.12	0.02
*Candida albicans*	0.09	-0.01	-0.02	-0.09	-0.22	0.24	0.40	-0.20	0.32	0.38	0.32	0.18
